# Recent Advances in the Studies on Luotonins

**DOI:** 10.3390/molecules16064861

**Published:** 2011-06-14

**Authors:** Jing Lu Liang, Hyo Chang Cha, Yurngdong Jahng

**Affiliations:** College of Pharmacy, Yeungnam University, Gyeongsan 712-749, Korea

**Keywords:** luotonin A, luotonin B, luotonin C, luotonin D, luotonin E, luotonin F, topoisomerase I, topoisomerase II

## Abstract

Luotonins are alkaloids from the aerial parts of *Peganum nigellastrum* Bunge. that display three major skeleton types. Luotonins A, B, and E are pyrroloquinazolino-quinoline alkaloids, luotonins C and D are canthin-6-one alkaloids, and luotonin F is a 4(3*H*)-quinazolinone alkaloid. All six luotonins have shown promising cytotoxicities towards selected human cancer cell lines, especially against leukemia P-388 cells. Luotonin A is the most active one, with its activity stemming from topoisomerase I-dependent DNA-cleavage. Such intriguing biological activities and unique structures have led not only to the development of synthetic methods for the efficient synthesis of these compounds, but also to interest in structural modifications for improving the biological properties. Recent progress in the study of luotonins is covered.

## 1. Introduction

The plant *Peganum nigellastrum* Bunge (Zygophyllaceae) has long been used in Chinese traditional medical practice for the treatment of rheumatism, abscesses, and diseases accompanying inflammation [1]. The basic fractions of *P. nigellastrum* showed potent anti-tumor activity [2], and the origin of such activity was revealed by identifying its constituents luotonin A (**1aa**) (We have two systems that one varies a substituent on the ring A-E (**1a**-**1x**) and the other varies the length of the bridge on the ring C (**1aa**-**1ac**) as seen in page 4875) and B (**1b**), which inhibited the growth of leukemia P-388 cells (IC_50_ of luotonin A = 1.8 μg/mL) [3]. Later, additional luotonins such as luotonin C (**2a**) and D (**2b**) [4] and luotonin E (**1c**) and F (**3**) [5] were consecutively isolated from the same plant. Most of these luotonins show promising cytotoxicity against leukemia P-388 cells [6,7,8] (see Table 1).

**Table 1:** Cytotoxic activity of luotonins against mouse leukemia P-388 cells [6]. 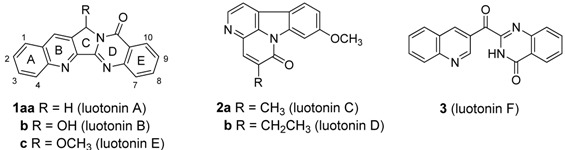

Compound	Luotonin A	Luotonin B	Luotonin C	Luotonin D	Luotonin E	Luotonin F
IC_50_ (μg/mL)	1.8	5.0	-	-	9.0	2.3

**Figure 1:**
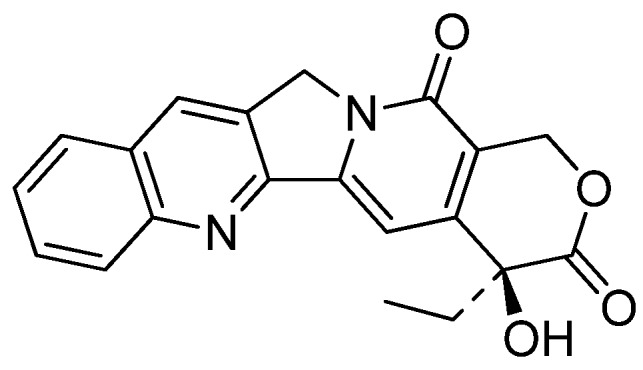
Structure of 20(*S*)-Camptothecin.

The mechanism of action of such cytotoxicity was first revealed by Hecht and his coworkers [9]. Luotonin A stabilizes the human DNA Topo I-DNA covalent binary complex by binding through the minor groove [10] and mediates topoisomerase I (Topo I)-dependent cytotoxicity in intact cells (IC_50_ = 5.7–12.6 μg/mL), like camptothecin (CPT, Figure 1) [11], that is a selective inhibitor of DNA Topo I [12,13,14,15]. Although the biological activities of luotonins towards selected human cancer cell lines are less potent and less selective to Topo I than CPT, the structural similarities of luotonin A, B, and E to CPT are enough to make them good lead compounds for the development of new potential anticancer agents. 

More than a decade has passed since the first discovery of luotonin A and B, yet only two review papers have appeared in this time, one by Ma *et al*. in 2005 [16] and another by Huang *et al*. [17] in 2009, focusing on the synthetic strategies for the total synthesis of luotonin A and covering some thirty publications. Michael’s annual reviews covering quinoline, quinazoline, and acridine alkaloids in *Natural Products Reports*, have also included mentions of the annual progress in the studies on luotonins since 1999 [18]. A *SCIFinder* literature search revealed 64 papers covering the six luotonins A-F. After the first review, more than 25 papers have been published, most of which cover the development of synthetic methods for the total synthesis and the structural modification of luotonin A. The present review covers roughly in chronological order the total synthesis of all six luotonins based on the synthetic strategies for the ring formation and the structure-biological activity relationship studies.

## 2. Synthesis

The six luotonins can be classified into three categories: luotonins A, B, and E are pyrrolo-quinazolinoquinoline alkaloids, luotonin F is a 4(3*H*)-quinazoline alkaloid, and luotonins C and D are canthin-6-one alkaloids. Six synthetic strategies have been employed for the synthesis of luotonins A, B, and E, including *via* formation of the pyridine core (ring B), the pyrrole core (ring C), the pyrimidinone core (ring D), the 5*H*-pyrrolo[4,3-*b*]pyridine core (rings B and C), the pyrrolo[1,2-*a*]pyrimidin-4(6*H*)-one core (rings C and D), and the pyrido[2’3’:3,4]pyrrolo[1,2-*a*]pyrimidin-4(6*H*)-one core (rings B,C,D) as the final step. 

### 2.1. via Formation of the Pyridine Core

**Scheme 1:**
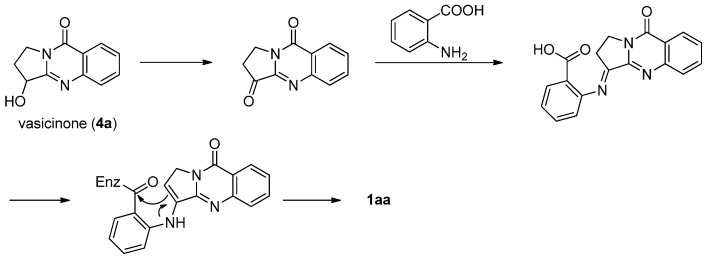
Hypothetical biosynthesis of luotonin A from vasicinone.

This strategy was applied in the first total synthesis of luotonins A and B. Ma *et al*. also isolated vasicinone (**4a**), a pyrroloquinazoline alkaloid from *Peganum nigellastrum* Bunge, along with luotonin A and some other alkaloids, which allowed them to propose a possible biosynthetic route to luotonin A from vasicinone (**4a**) and anthranilic acid as shown in Scheme 1 [3]. 

**Scheme 2:**
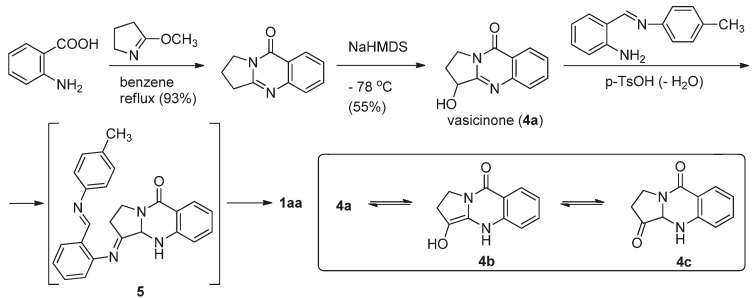
Ma *et al*.’s [19] synthesis of luotonin A.

A few years later, the same authors used the reaction between **4a** and *N*-(2-aminobenzylidene)-*p*-toluidine in the presence of *p*-TsOH to prepare luotonin A [19] (Scheme 2). The starting vasicinone can be tautomerized to the corresponding keto tautomer **4c**, which can be readily condensed with the toluidine derivative of 2-aminobenzaldehyde to form a Schiff’s base **5** which then undergoes cyclization *via* an enamine, followed by aromatization to afford luotonin A.

**Scheme 3:**
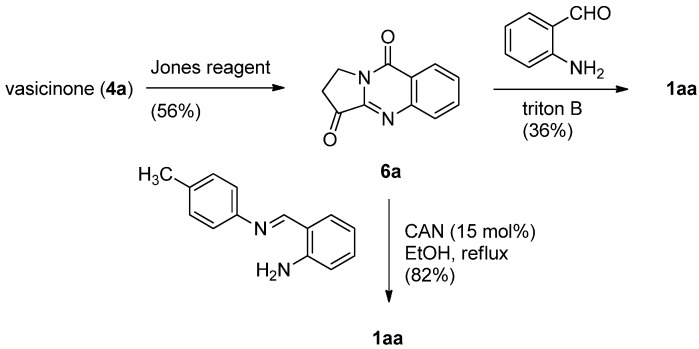
Kelly *et al*.’s [23] synthesis of lutonin A.

Since its discovery in 1882 the Friedländer condensation of 2-aminobenzaldehyde and ketones has long been one of the most efficient synthetic methods for preparing quinolines [20,21,22]. Kelly *et al*. applied the Friedländer condensation to pyrrolo[2,1-*b*]quinazoline-3,9-dione (**6a**), prepared by Jones oxidation of vasicinone, to yield luotonin A in 36% yield (Scheme 3) [23,24]. Recently, this low yield was increased to 66% by employing cerium(IV) ammonium nitrate (CAN) as a catalyst [25]. In addition, replacement of 2-aminobenzaldehyde by *N*-(2-aminobenzylidene)-*p*-toluidine improved the yield up to 82%.

**Scheme 4:**
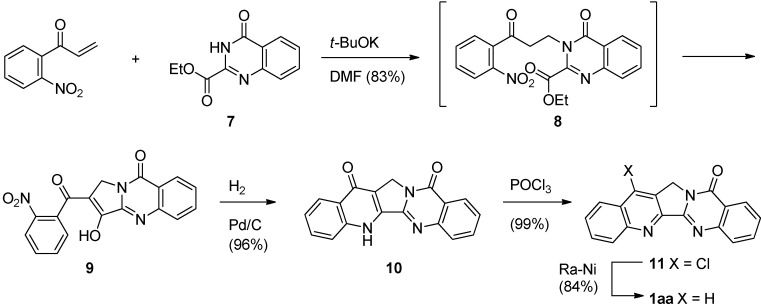
Mason and Bergman’s [26] synthesis of luotonin A.

In addition, the quinoline ring can be formed *via* 4(1*H*)-quinolinone [26]. Michael addition of ethyl 4-oxo-3,4-dihydroquinazoline-2-carboxylate (**7**) to 1-(2-nitrophenyl)propenone gave an 83% yield of compound **8**, which undergoes a spontaneous intramolecular Claisen condensation to afford enolizable 1,3-diketone **9** (Scheme 4). Catalytic hydrogenation of **9** in the presence of Pd/C afforded the 4(1*H*)-quinolinone derivative **10**, which was then chlorinated using POCl_3_ to yield 14-chloroluotonin A (**11**). Hydrogenolysis of **11** with activated Ra-Ni gave luotonin A. 14-Cloroluotonin A can be used as a useful starting material for introducing various substituents at the C14 position of luotonin A.

### 2.2. via Formation of the Pyrrole Core

**Scheme 5:**
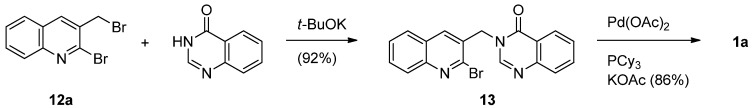
Harayama *et al*.’s [27,28] synthesis of luotonin A.

Harayama *et al*. reported the first synthesis of luotonin A *via* C-ring formation, in which Heck biaryl coupling reaction was employed to form a C-C bond between C2 of a 4(3*H*)-quinazolinone and C2 of a quinoline using an electrophilic Pd reagent in DMF. *N*-Alkylation of 4(3*H*)-quinazolinone with 2-bromoquinolin-3-ylmethyl bromide yielded 3-[(2-bromoquinolin-3-yl)methyl]-4(3*H*)-quinazolinone (**12a**) which was then subjected to Heck coupling condition [27,28] (Scheme 5). 

**Scheme 6:**
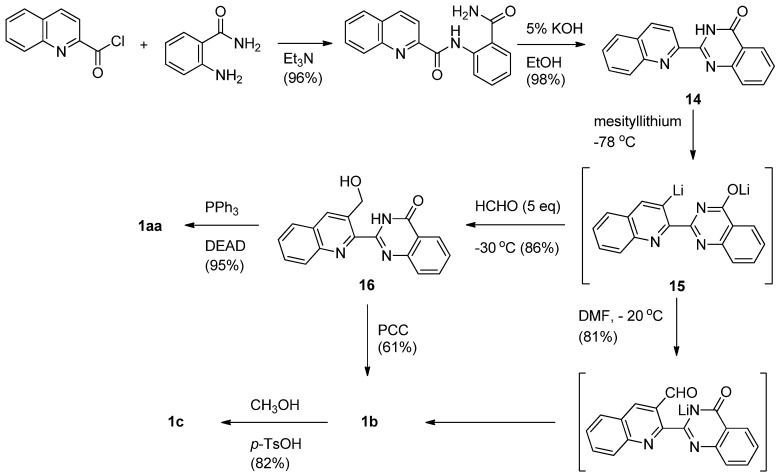
Mhaske and Argade’s [29] synthesis of luotonin A.

On the other hand, Mhaske and Argade employed a regioselective quinazolininone-directed *ortho*-lithiation on an adjacent quinoline moiety as a key step to make luotonins A, B and E [29] (Scheme 6). Acylation of anthranilamide with quinaldic acid chloride afforded the corresponding diamide, which was readily cyclized by 5% KOH to yield 2-quinolino-4(3*H*)-quinazolinone (**14**) in quantitative yield. Lithiation of **14** afforded the di-lithiated species **15** which can be readily converted to luotonin A in two steps *via* 3-hydroxymethyl derivative **16** employing a Mitsunobu condensation. Reaction of **15** with DMF spontaneously afforded luotonin B, which could also be prepared by PCC oxidation of **16**. Luotonin B was converted to luotonin E by treatment with methanol in the presence of *p*-TsOH. The procedure has the advantage of generating a library of quinazolinone alkaloids by reacting di-lithiated species with a variety of electrophiles. 

**Scheme 7:**
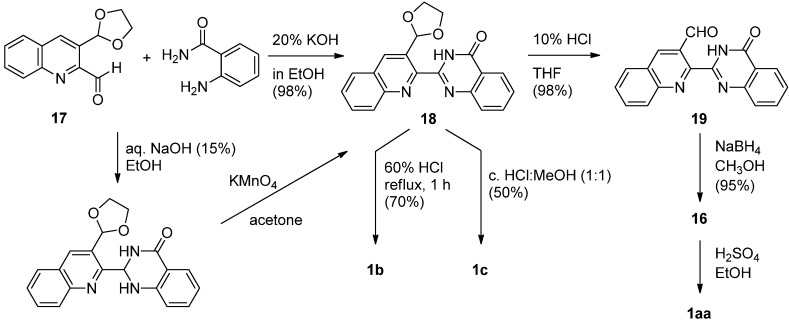
Chavan and Sivappa’s [30] synthesis of luotonin A.

Intermediate **16** was also be prepared by NaBH_4_ reduction of the corresponding carbaldehyde **19**, prepared by hydrolysis of the prerequisite acetal **18** by Chavan and Sivappa [30] (Scheme 7). The key acetal **18** was prepared either directly from quinoline-2-carbaldehyde **17** and anthranilamide in the presence of 20% KOH in EtOH or by a two-step synthesis employing 15% NaOH in EtOH followed by oxidation with KMnO_4_. It is noteworthy that this procedure afford three luotonins from a common intermediate **18**. Hydrolysis of the acetal moiety in **18** and reduction of the resulting aldehyde afforded the corresponding alcohol 16 which could be cyclized either by a Mitsunobu reaction or, more conveniently, by treatment with sulfuric acid in EtOH to yield luotonin A. Alternatively, refluxing of **18** in aqueous acid resulted in luotonin B (**1b**) while refluxing in a 1:1 mixture of conc. HCl-CH_3_OH afforded luotonin E (**1c**). 

### 2.3. via Formation of the Pyrimidinone Core

Among a variety of available synthetic procedures for assembling the pyrimidinone nucleus, Kametani’s one has long been employed for the construction of various quinazolinone derivatives from lactams and anthranilic acid in the presence of thionyl chloride [31]. Such a method has been applied with modifications to the synthesis of luotonin A in several cases. 

**Scheme 8:**
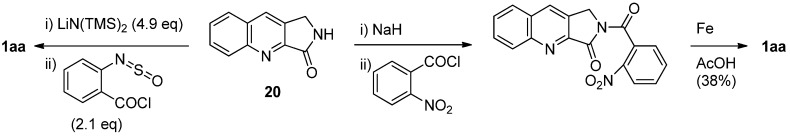
Kametani’s [31] method for the synthesis of luotonin A and its modification.

**Scheme 9:**
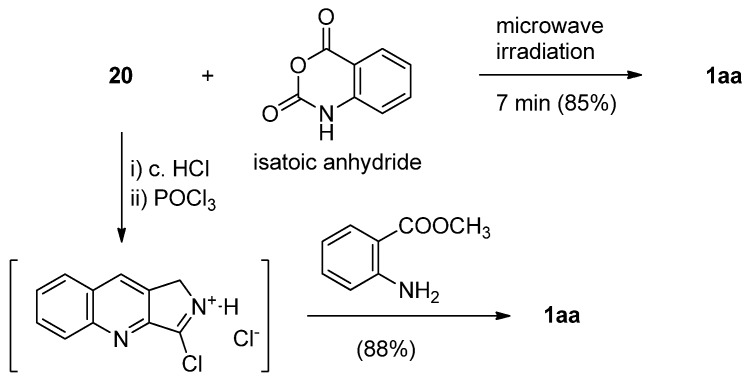
Modified Kametani’s synthesis of luotonin A.

In fact, the first total synthesis of luotonin A was achieved by Wang and Ganesan, in which 2,3-dihydro-[1*H*]-pyrrolo[3,4-*b*]quinolin-3-one (**20**) was coupled with 2-sulfinylaminobenzoyl chloride, generated from anthranilic acid and thionyl chloride, in the presence of LiN(TMS)_2_[32] while direct condensation of the lactam and 2-sulfinylaminobenzoyl chloride by Kametani’s quinazoline formation procedure yielded only a 6% yield of luotonin A (Scheme 8). In addition, reaction of lactam **20** with 2-nitrobenzoyl chloride followed by reduction of the nitro group resulted in luotonin A in 38% overall yield [33]. Microwave assisted synthesis of the quinazoline ring from a lactam and isatoic anhydride was also applied to the synthesis of luotonin A (Scheme 9). Thus, a mixture of lactam **20** and isatoic anhydride was subjected to microwave irradiation at 450 watts for 7 minutes to yield luotonin A in 85% yield [34]. On the other hand, direct reaction of **20** and methyl anthranilate in the presence of POCl_3_ yielded luotonin A [35,36], while the use of the HCl salt of **20** improved the yield significantly [37]. Such reaction conditions have been further simplified in a one-pot reaction of lactam **20**, ethyl anthranilate, and SOCl_2_ in refluxing benzene to afford luotonin A in 75% yield [38]. 

### 2.4. via Formation of the Pyrrolo[4,3-b]pyridine Core

**Scheme 10:**
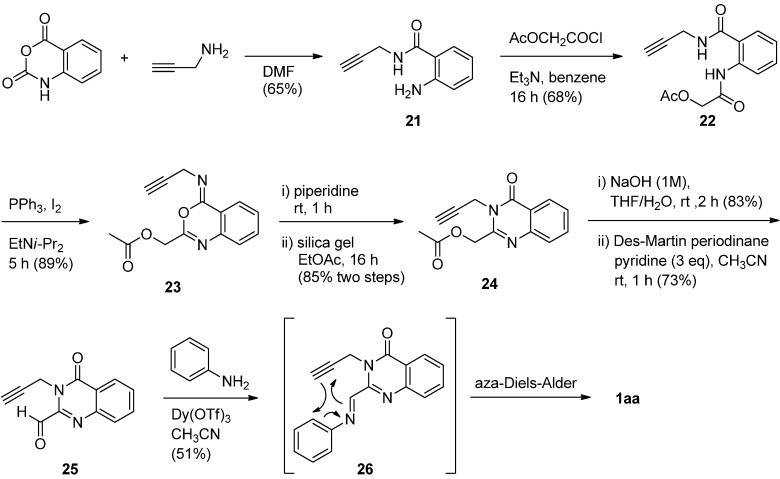
Twin and Batey’s [39] synthesis of luotonin A.

Twin and Batey employed an intramolecular hetero Diels-Alder (Povarov) reaction to construct the quinoline and pyrrole nuclei in one step from 2-formyl-3-propargyl-4(3*H*)-quinazolinone (**24**) and aniline in the presence of the mild Lewis acid catalyst Dy(OTf)_3_ [39]. The coupling reaction proceeded through sequential imine formation between a formyl group and aniline and a formal intramolecular aza-Diels-Alder reaction to form luotonin A (Scheme 10). Propargyl amide **21**, readily prepared by refluxing isatoic anhydride and propargylamine, was acylated with 2-acetyloxyacetyl chloride to form the corresponding amide **22**, which was subjected to Mazurkiewicz’ synthesis and rearrangement of 4-imino-4*H*-3,1-benzoxazine by triphenylphosphine and iodine in the presence of Hünig’s base to yield **23**. A two-step, one-pot rearrangement of **23** using piperidine followed by treatment with silica gel afforded 2-acetyloxymethyl-3-propargyl-4(3*H*)-quinazolinone (**24**) in 85% yield. Base-catalyzed hydrolysis of **24** followed by Dess-Martin periodinane oxidation gave the desired **25**. Reaction of **25** with aniline afforded the corresponding Schiff’s base **26** which then undergoes the intermolecular Povarov reaction in the presence of Dy(OTf)_3_to form luotonin A *via* an intramolecular inverse-electron demand hetero Diels-Alder reaction. Although the yields of all the reaction steps are good, overall the presented method suffers from a lengthy synthetic sequence starting from commercially available isatoic anhydride.

**Scheme 11:**
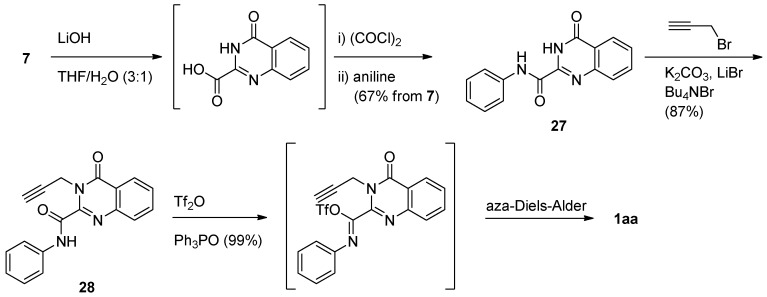
Yao *et al*.’s [40] synthesis of luotonin A.

A similar cascade reaction was applied to **27** in the presence of bis(triphenyl)oxodiphosphonium triflate (prepared *in situ*) to yield luotonin A *via* an intramolecular aza Diels-Alder reaction [40] (Scheme 11). Hydrolysis of ethyl 4-oxo-3,4-dihydroquinazoline-2-carboxylate (**7**), followed by a two-step amide formation, yielded the corresponding *N*-phenyl-4(3*H*)-quinazoline-2-carboxamide (**27**). *N*-phenyl-3-propargyl-4(3*H*)-quinazoline-2-carboxamide (**28**) was treated with triphenylphosphine oxide in the presence of (CF_3_SO_2_)_2_O to yield luotonin A in 58% overall yield.

**Scheme 12:**
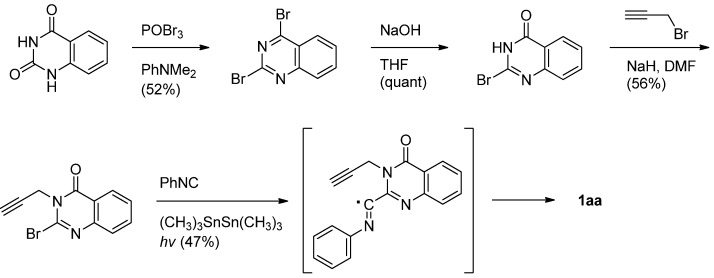
Curran *et al*.’s [41] synthesis of luotonin A.

Curran and his coworkers employed a similar intramolecular radical coupling of 2-bromo-3-propargyl-4(3*H*)-quinazolinone and phenyl isonitrile in the presence of hexamethylditin and light. [41] (Scheme 12). 

**Scheme 13:**
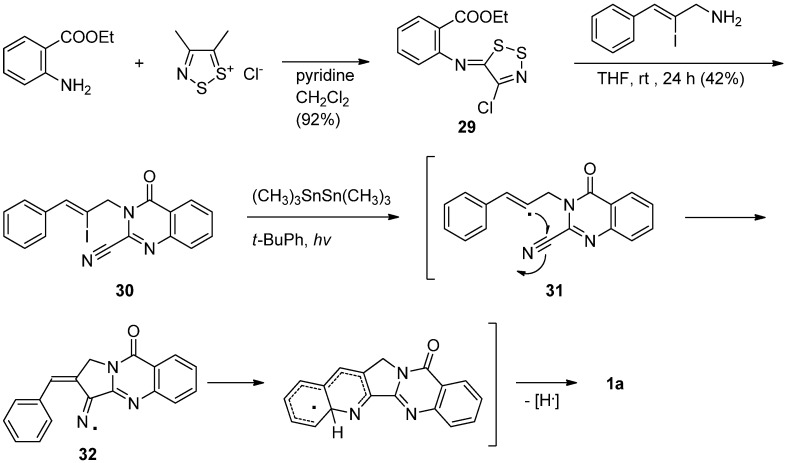
Bowman *et al*.’s [42,43] synthesis of luotonin A.

Bowman *et al*. have reported yet another versatile synthesis employing a cascade radical cyclization of a cyano-alkene compound to produce luotonin A [42,43] (Scheme 13). The prerequisite radical precursor **30** was prepared in 42% yield by a reaction of ethyl 2-[(4-chloro-5*H*-1,2,3-dithiazol-5-yliden)amino]-benzene-1-carboxylate (**29**) and (*Z*)-2-iodo-3-phenylprop-2-en-1-amine while a direct *N*-alkylation of 2-cyano-4(3*H*)-quinazolinone with (*Z*)-2-iodo-3-phenylprop-2-enyl bromide yielded **30** in 16% yield. Treatment of above vinyl iodide **30** with hexamethylditin in the presence of light yielded the corresponding radical **31** which undergoes 5-*exo* cyclization onto the nitrile group at C2 of the 4(3*H*)-quinazolinone nucleus to produce the iminyl radical intermediate **32**, which subsequently undergoes a homolytic aromatic substitution *via* 6-endo (or 5-*exo* cyclization followed by neophyl rearrangement) with the loss of a hydrogen atom to yield luotonin A.

### 2.5. via Formation of the Pyrrolo[1,2-a]pyrimidin-4(6H)-one Core

**Scheme 14:**
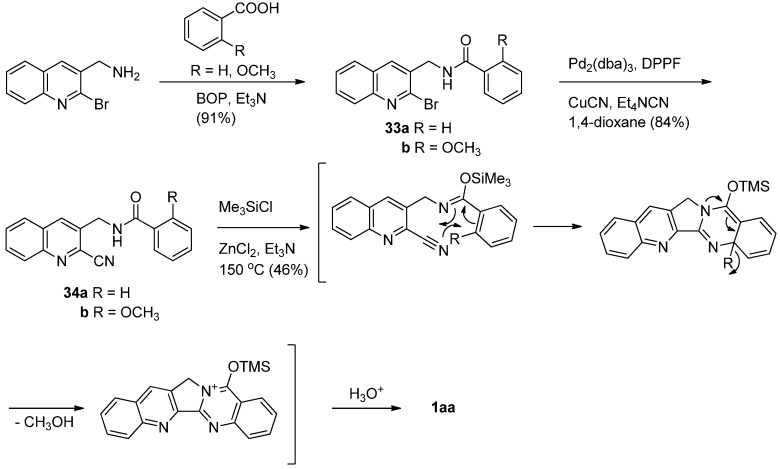
Toyota *et al*.’s [44,45] synthesis of luotonin A.

Toyota *et al*. reported an additional efficient synthesis of luotonin A employing an intramolecular hetero Diels-alder reaction of an aryl iminoether with an aryl nitrile [44,45] (Scheme 14). Palladium-catalyzed coupling reaction of 2-bromoquinoline derivatives **33** with CuCN afforded the corresponding 2-cyanoquinoline derivatives **34**. Heating **34b** with TMSCl and Et_3_N at 150 °C in toluene in a stainless sealed tube in the presence of ZnCl_2_, afforded luotonin A in 46% yield, while 34a did not yield luotonin A. The methoxy group in **34b** apparently not only increases the HOMO energy to accelerate the reaction, but also readily acts as a good leaving group after cyclization to restore the aromaticity of the benzene ring (ring E). 

**Scheme 15:**
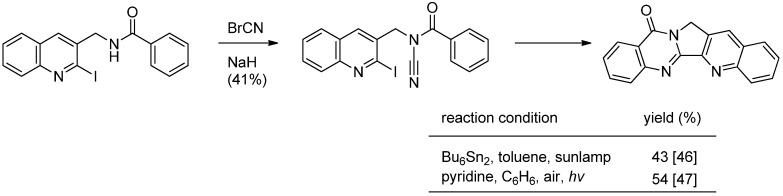
Courillon *et al*.’s [46,47] synthesis of luotonin A.

Courillon and coworkers reported a cascade radical cyclization of *N*-acylcyanamides as an alternative procedure for the 4(3*H*)-quinazolinone nucleus, which led to the total synthesis of luotonin A [46,47] (Scheme 15). Cyanation of *N*-[(2-iodoquinol-3-yl)methyl]benzamide with cyanogen bromide afforded the corresponding *N*-CN compound which was subjected to radical cyclization to yield luotonin A. The yields were highly dependent on the reaction conditions.

**Scheme 16:**
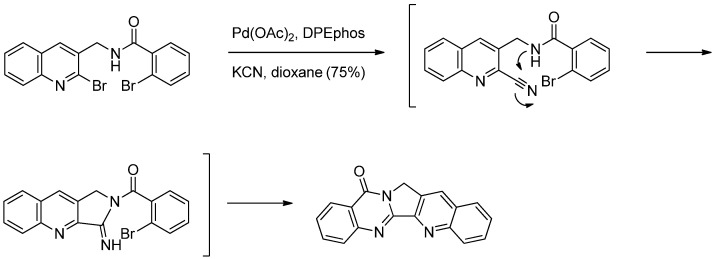
Li *et al*.’s [48] synthesis of luotonin A.

This method has been modified by Ju *et al*. who employed sequential cyanation, *N*-addition, followed by *N*-arylation of *N*-[(2-bromoquinolin-3-yl)methyl]-2-bromobenzamide leading to construction of C- and D-rings in one step in a two-stage, one-pot manner [48] (Scheme 16). The reaction was initiated by the Pd-catalyzed cyanation at C2 of the quinoline moiety, followed by intermolecular nucleophilic *N*-addition of an amide to a nitrile to generate an imidamide intermediate, which undergoes additional Pd-catalyzed intramolecular *N*-arylation with an aryl halide to allow the one-step construction of the pyrroloquinazolinone skeleton. 

### 2.6. Formation of Rings B, C and D

**Scheme 17:**
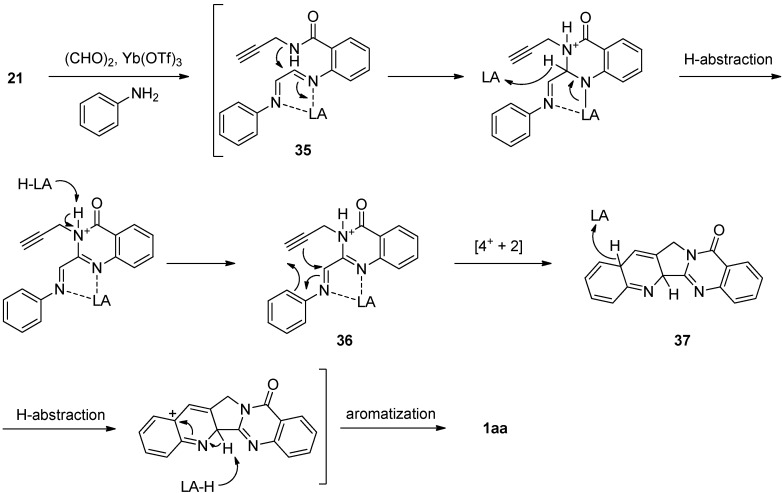
Chu *et al*.’s [49] synthesis of luotonin A.

The cascade cyclization method was recently modified by Tseng *et al.* The reaction of **21** with glyoxal in the presence of Yb(OTf)_3_yielded 1,4-diazadiene **35** which underwent cyclization, followed by subsequent hydrogen abstraction to yield *N*-phenyliminium azadiene **36** [49] (Scheme 17). Lewis acid-catalyzed inverse electron-demand aza-Diels-Alder [4+ + 2] cycloaddition between *N*-chelated *N*-phenyliminium azadiene and the electron-rich dienophile in **36** afforded pentacyclic system **37** which in turn provided luotonin A by hydrogen abstraction followed by aromatization.

### 2.7. Conversion of Luotonin A to Luotonins B and E

**Scheme 18:**
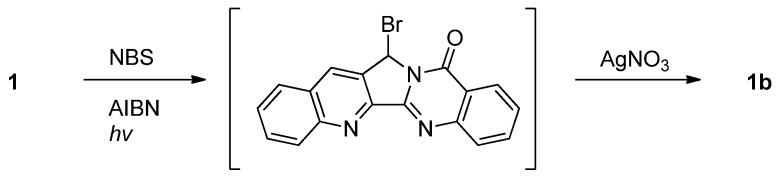
Harayama *et al*’s [27,28] synthesis of luotonin B.

Luotonin A underwent slow conversion to luotonin B when a solution of the compound in chloroform was exposed to sunlight for two weeks [3]. Luotonin A can also be converted to luotonin B in two steps by AIBN-catalyzed bromination at the benzylic position followed by Ag(I)-mediated hydroxylation [27,28] (Scheme 18).

**Scheme 19:**
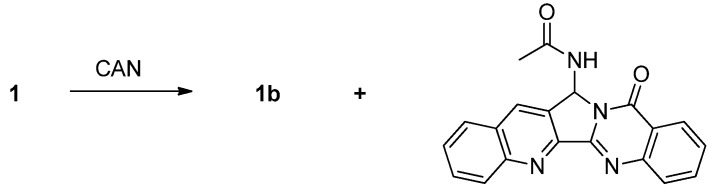
Ma *et al*.’s [6] synthesis of luotonin B.

Oxidation of luotonin A with CAN afforded luotonin B along with 14-acetamidoluotonin A, of which the reaction mechanism had been suggested [6] (Scheme 19).

**Scheme 20:**
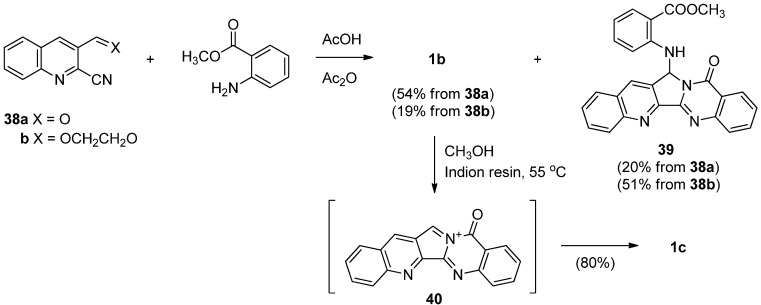
Kumar *et al*.’s [50] synthesis of luotonins B and E.

Very recently, Wagh *et al.* reported a concise and convergent synthesis of luotonin B in 54% and 19%, respectively, along with **39**, by employing a cascade cyclization of 2-cyanoquinoline-3-aldehyde (**38a**) or 3-(1,3-dioxolan-2-yl)quinoline-2-carbonitrile (**38b**) with methyl anthranilate in a mixture of acetic acid and acetic anhydride [50] (Scheme 20). Dehydration of luotonin B by Indion resin or Amberlist 25 acidic resin resulted in imminium ion **40** which was then underwent methylation to give luotonin E in 80% yield.

## 3. Synthesis of Luotonin C and D

**Scheme 21:**
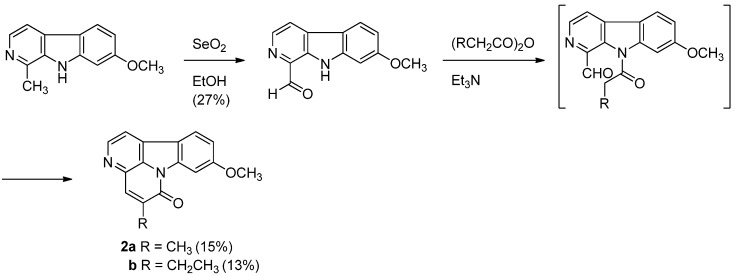
Ma *et al*.’s [4] synthesis of luotonins C and D.

Although numerous synthetic procedures for the parent canthin-6-one core have been reported [51,52,53,54], only one synthetic procedure for luotonin C and D has been published [4]. Luotonin C and D were first prepared in two steps by a biomimetic way from harmine coexisting with these alkaloids in the same plant source. Selenium oxidation of harmine afforded the corresponding aldehyde in 27% yield which was acylated with propionic anhydride and *n*-butyric anhydride in the presence of triethylamine to yield luotonin C and D in 15% and 13% yields, respectively (Scheme 21). 

## 4. Synthesis of Luotonin E and F

**Scheme 22:**
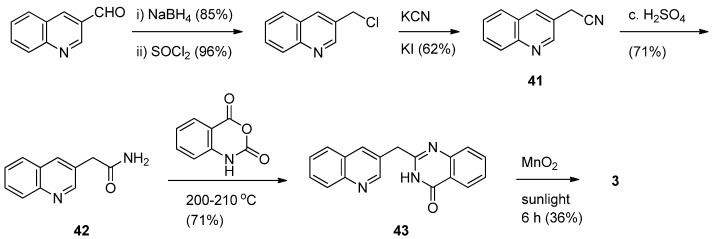
Ma *et al*.’s [5] synthesis of luotonin F.

A synthetic sequence of Nomura and his coworkers for luotonin F started from 3-formylquinoline [5]. 3-Hydroxymethylquinoline prepared by a reduction of 3-formylquinoline with NaBH_4_ was converted to the corresponding 3-chloromethylquinoline which was then subjected to nucleophilic substitution by KCN to provide the nitrile compound **41**. Partial hydrolysis by conc. sulfuric acid provided an amide **42** which was condensed with isatoic anhydride to yield 2-(quinolin-3-ylmethyl)-quinazolin-4(3*H*)-one (deoxyluotonin F, **43**). The synthetic sequence was completed by oxidation of **43** with activated MnO_2_ in the presence of sunlight to afford luotonin F in 36% yield (Scheme 22).

**Scheme 23:**
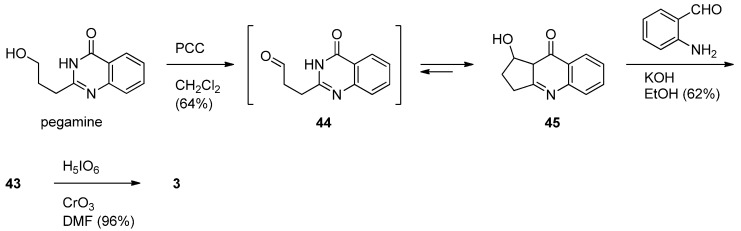
Mhaske and Argade’s [55] synthesis of luotonin F.

Mhaske and Argade described a biogenetic synthesis of luotonin F starting from pegamine [55]. PCC oxidation of pegamine afforded isovasicinone (**45**) *via* the corresponding aldehyde **44**which was subjected to Friedländer condensation with 2-aminobenzaldehyde in the presence of ethanolic KOH to give deoxyluotonin F in 62% yield *via in situ* ring opening of **45**. Chromium trioxide catalyzed periodic acid oxidation of deoxyluotonin F afforded the desired luotonin F in 96% yield (Scheme 23).

## 5. Structural Modifications and Structure-Activity Relationships

Although luotonin A is less potent as well as less efficient than camptothecin in its ability to stabilize the covalent complex between DNA and Topo I, the structural similarities have spurred not only efforts to introduce a variety of substituents, but also to design and synthesize related compounds to improve such activities. Up to now, 10 papers pursuing structure-activity relationship study focusing a modification on rings A, B, C, D, and E have been published, covering inhibitory activities against DNA Topo I and/or DNA topoisomerase II (Topo II). It should be noted that the inhibitory activities of luotonin A (IC_50_ = 28.5 μM) and F against Topo II were somewhat related to their cytotoxicities [6,56].

### 5.1. Modifications on Ring A

The introduction of a substituent on ring A significantly affects the cytotoxicity. Compound **1d** with fluorine at C3 showed strong inhibitory activity against Topo I, comparable to that of CPT, which was not directly related to its cytotoxicity. The compounds **1h** and **1i** with *N*,*N*-diethylaminoethoxy and *N*,*N*-dimethylaminoethoxy groups showed improved cytotoxicity against HCT-116 cancer (human colon tumor) cell lines [57]. It should be noted that stronger cytotoxicity of **1f** against H460 (human non-small lung carcinoma cell line) by Topo-I-mediated DNA cleavage may open a new vista for future structural modification on luotonins [58]. On the other hand, compound **1k** with an ethyl group at C14 displayed slightly improved cytotoxicity compared to that of luotonin A [30]. In addition, 14-trifluoromethylluotonin A (**1m**) caused apoptosis of cultured colon adenocarcinoma and leukemia cells (IC_50_ = 17(3) and 21(4) μM, respectively), which also showed inhibitory activity against Topo I at 40 μM/L [59]. These data are summarized in Table 2.

### 5.2. Modifications on Ring C

Structure-activity relationship studies on camptothecin and related compounds revealed that introduction of various substituents such as alkyl, alkylamino and alkylimino groups on the C4-position of quinoline moiety improved the Topo I inhibitory activity. Based on this, a series of compounds with a substituent on the ring C have been introduced. However, no detailed studies on their biological properties were described [26].

**Table 2:** Inhibitory activities against Topo I and cytotoxicities of derivatives of luotonin A on ring A and B. 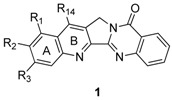

Compd	R_1_	R_2_	R_3_	R_14_	Inhibitory activity against Topo I [% (rel. activity)] at 100 μM	IC_50_ (μM)		
						HCT-116	HL-60	H460
**1aa**	H	H	H	H	33.7 (0.40) [56]	51.11 [56]	>100 [56]	7.7 [58] ^a)^
**1d**	H	H	F	H	81.1 (0.91) [56]	>100 [56]	56.65 [56]	-
**1e**	H	OH	H	H	54.1 (0.65) [56]	56.6 [56]	66.29 [56]	-
**1f**	H	OMe	OMe	H	-	-	-	5.47 [58]
**1g**	H	-OCH_2_CH_2_O-		H	19.2 (0.23) [56]	19.36 [56]	21.78 [56]	81 [58]
**1h**	H	R ^c)^	H	H	-	-	2 [40]	-
**1i**	H	R ^d)^	H	H	-	-	2 [40]	-
**1j**	OH	R ^e)^	H	H	-	-	10 [40]	-
**1k**	H	H	H	Et	slightly more active ^b)^[41]	-	-	-
**1l**	H	OH	H	Et	similar to luotonin A [41]	-	-	-
**1m**	H	H	H	CF_3_	inhibition at 40 μM [59]	-	-	-
Doxo ^f)^					-	2.31 [56]	4.78 [56]	-
CPT					83.9 (1) [56]	2.17 [56]	5.51 [56]	-

^a)^ Values obtained from 72 h culture, ^b)^ no detailed data were available [41], ^c)^ R = OCH_2_CH_2_NEt_2_, ^d)^ OCH_2_CH_2_NMe_2_, ^e)^ CH_2_NMe_2_, ^f)^ doxorubicin.

**Scheme 24:**
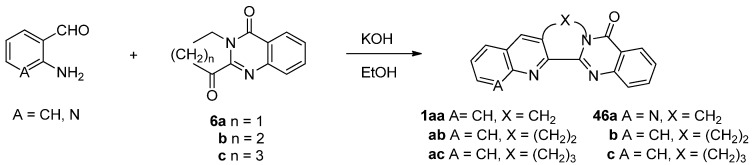
Jahng *et al*.’s [60] synthesis of luotonin A homologues and their aza-derivatives.

On the other hand, the Friedländer condensation of lactam **6a** and its homologues with 2-aminobenz-aldehyde and 2-aminonicotinaldehyde provided a homologous series of luotonin A [60] and their aza-derivatives, respectively (Scheme 24). 

The cytotoxicity of the compounds decreased as the length of bridge increased and a system with 1,8-naphthyridine generally showed increased cytotoxicity, especially towards human cancer cell lines. 7-Azaluotonin A (**46a**) showed the strongest cytotoxicity against L1210 (leukemia tumor) (IC_50_ = 0.13 μM), A549 (lung carcinoma) (IC_50_ = 6.3 μM) and SK-OV-3 (ovary adenocarcinoma) (IC_50_ = 5.6 μM) which may imply a direction of future study for development of anticancer agents based on luotonin A. 

### 5.3. Modifications on Rings D and E

Studies of Hecht and his coworkers [35,36] and Dallavalle and her coworkers [58] have focused on the introduction of substituents on ring E and the substitution of ring A by its isosteres employing a reaction of **21** with appropriate 2-aminoarenecarboxylic acids, of which the parts are summarized in Table 3. 

**Table 3:** Cytotoxicities of derivatives of luotonin A on ring E and isosteres. 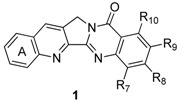

	R_7_	R_8_	R_9_	R_10_	IC_50_ (μM)				
					Galactose [25]	ADR-Res [40]	HeLa [40]	MCF7 [40]	H460 [41] ^a)^
**1aa**	H	H	H	H	0.58	5	3	3	7.7
**1n**	H	H	F	H	9.58	-	-	-	-
**1o**	H	NH_2_	H	H	15.7	-	-	-	-
**1p**	H	H	H	Cl	-	-	-	-	13
**1q**	H	H	NO_2_	H	-	-	-	-	23
**1r**	CH_3_	H	H	H	-	-	-	-	7
**1s**	H	H	Cl	H	-	-	-	-	45
**1t**	H	H	OH	H	-	-	-	-	3.8
**1u**	Cl	H	H	H	-	13	>10	10	-
**1v**	NH_2_	H	H	H	-	3	2	2	-
**1w**	OCH_3_e	H	H	H	-	>20	12	>20	11
**1x**	H	OH	OH	H	-	>20	6	10	-
**46**					11.8	-	-	-	-
**47**					91.4	-	-	-	-
CPT					0.86	0.01	0.01	0.01	-
SN-38					-	-	-	-	0.21 ^b)^

^a)^ Values obtained from 72 h culture. ^b)^ Values from 1 h culture.

**Figure 2:**
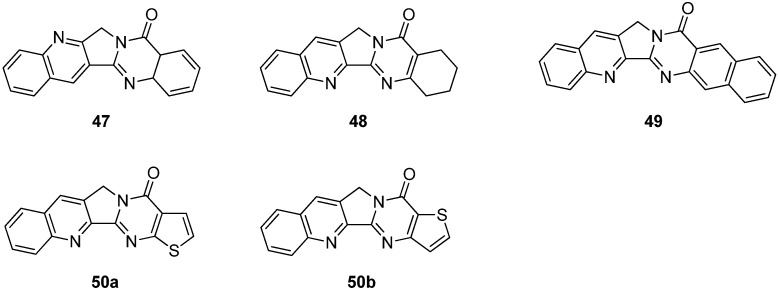
Isosteres of luotonin A.

Although decisive conclusions about the structure-activity relationships cannot be drawn, a couple of significant features can be deduced. The activities of a compound with an amino group at C7 (**1v**) were increased against a broad range of selected human cancer cell lines such as ADR-Res (adriamycin-resistant breast carcinoma), HeLa (cervical carcinoma), MCF7 (breast carcinoma) and H460 compared to luotonin A. The compound with a hydroxyl group at C9 (**1t**) also showed promising cytotoxicity against the HL460 cancer cell line. Introduction of a substituent at C3 (**1n,o**) is also important for maintaining such cytotoxic activities. Compounds **1n** and **1o** showed reasonably strong Topo-I dependent cytotoxicity toward a yeast strain grown on galactose medium with IC_50_ values of 9.58 and 15.7 μM, respectively. It should be noted that compound **47**, a regioisomer of luotonin A (Figure 2), showed promising activity (11.8 μM) while reduction of the A-ring (*i.e*., compound **48**, Figure 2) reduced activity significantly. Although system with an isosteric aromatic ring including naphthalene and thiophene units (**49 **and **50**, respectively, Figure 2) strongly stabilized the Topo I-DNA binary complex, these isosteres did not show any promising activity against human cancer cell lines [36] implying that ring A should be a benzene moiety for better cytotoxicity. Furthermore the stability of the Topo I-DNA binary complex might not be directly related to cytotoxicity. 

### 5.4. 14-Azacamptothecin – A Hybrid Molecule of Luotonin A and Camptothecin

**Scheme 25:**
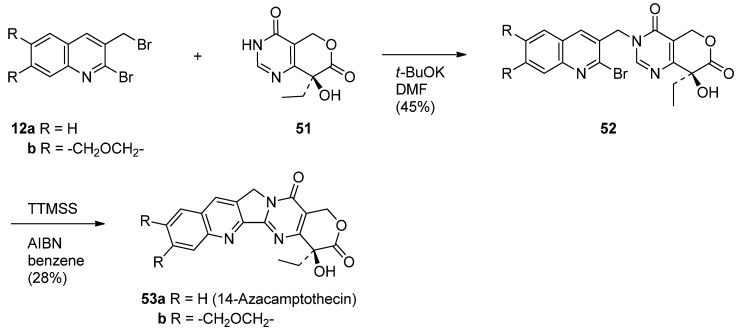
Synthesis of 14-azacamptothecin and its derivative.

Hecht and his coworkers designed 14-aza-camptothecin (**53a**) [61] and its methylenedioxy derivative (**53b**) [45] as a hybrid of camptothecin and luotonin A and prepared them in two-steps (*N*-alklyation followed by a radical-mediated cyclization) from 2-bromo-3-(bromomethyl)quinoline (**12a**) and its 6,7-methylenedioxy derivative (**12b**) and pyranopyrimidindione (**51**) (Scheme 25). 

**Scheme 26:**
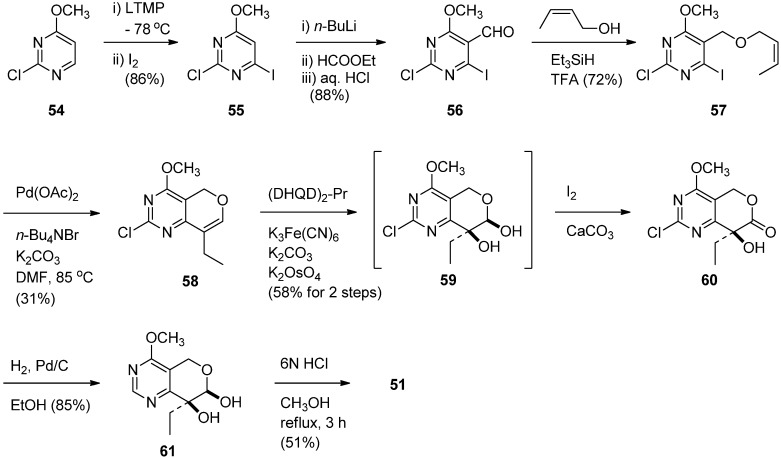
Synthesis of chiral synthon of 14-azacamptothecin.

The prerequisite **51** was prepared in nine steps from commercially available 2-chloro-6-methoxy-pyrimidine [60] (Scheme 26). Iodination of **54** gave 4-iodopyrimidine **55** which was lithiated with *n*-BuLi and subsequently treated with ethyl formate to give the corresponding aldehyde **56** in 88% yield. Reductive etherification with crotonyl alcohol in the presence of Et_3_SiH and TFA afforded crotyl ether **57** which was subjected to the Heck coupling to yield an alkene **58**. Asymmetric dihydroxylation of **58** in the presence of the chiral ligand (DHQD)_2_-PYR (AD-system) afforded the corresponding lactol **59** which was not isolated, but instead treated with I_2_-mediated oxidation to give a lactone **60**. The lactone was dechlorinated to provide **61** by hydrogenolysis over 10% Pd/C and subsequent hydrolysis with 6N HCl to afford the desired **51** in 4.9% overall yield. 

Advantages of 14-aza-camptothecin and its methylenedioxy derivative **53b** lie not only in its improved water solubility in water and ability to inhibit Topo I-mediated DNA relaxation more efficiently than CPT or luotonin A, but also in its stabilization of the covalent binary complex (Table 4).

**Table 4:** Human Topo I dependent cytotoxicity of CPTs and luotonin A towards *Saccharomyces cerevisiae*.^a)^

Compound	Concentration (*μ*M)	Inhibition on growth medium (%)		Topo I dependent cytotoxicity (IC_50_, *μ*M)^b)^
		Raffinose	Galactose	
CPT	1.0	0	74	0.15
Luotonin A (**1aa**)	1.0	0	36	
	0.5	0	23	
14-aza-CPT (**53a**)	2.0	0	46	>10
**53b**				1.28

^a)^ Inhibition of RS321Nph-Topo I grown in minimal medium containing 3% raffinose or galactose for 2 days at 30 °C. ^b)^ Values were obtained against A549 cells using an MTT assay.

## 6. Conclusions and Perspectives

Increasing interest in the luotonins especially luotonin A (**1aa**) may stem from its inhibitory activity against topoisomerases and cytotoxicity against various cancer cell lines. Such intriguing properties and characteristic structure have led to 13 synthetic strategies for the total synthesis of luotonin A and several synthetic methods for luotonin B and E. However, a synthetic method that is generally able to introduce substituents on the rings more efficiently (*i.e.*, a method to introduce more than two substituents onto the different rings), has not as yet been established. Although the introduction of substituent(s) on the A, B, C, and E rings has been explored during last decade, the nature of the substituents as well as positions of the substituents on the rings are somewhat limited up to now due to the lack of synthetic methods of general applicability. In order to achieve the structural optimization of luotonin A and to improve the biological as well as pharmacokinetic properties as an anticancer drug, it will be necessary to synthesize more derivatives systematically. Studies on the development of CPT-related anticancer drugs [12,13,14,15,63] may afford useful information and a guideline for the future structural modification on luotonins.

## References

[1] Xiao P.-G. (1992). A Pictorial Encyclopaedia of Chinese Medical Herbs.

[2] Xiao X.-H., Qiu G.-L., Wang H.-L., Liu L.-S., Zheng R.-L., Jia Z.-J., Deng Z.-B. (1988). Effect of Alkaloids from *Peganum nigellastrum* on Mouse Ascitic Hepatoma and Isolated Cells. Chin. J. Pharmacol. Toxocol..

[3] Ma Z.-Z., Hano Y., Nomura T., Chen Y.-J. (1997). Two New Pyrroloquinazolinoquinoline Alkaloids from *Peganum nigellastrum*. Heterocycles.

[4] Ma Z.-Z., Hano Y., Nomura T., Chen Y.-J. (2000). Alkaloids and Phenylpropanoids from *Peganum nigellastrum*. Phytochemistry.

[5] Ma Z.-Z., Hano Y., Nomura T., Chen Y.-J. (1999). Two New Quinazoline-Quinoline Alkaloids from *Peganum nigellastrum*. Heterocycles.

[6] Ma Z., Hano Y., Nomura T., Chen Y. (2004). Novel Quinazoline-quinoline Alkaloids with Cytotoxicites and DNS Topoisomerase II Inhibitory Activities. Biorg. Med. Chem. Lett..

[7] Ma Z.-Z., Hano Y., Nomura T., Chen Y.-J. (1999). The Structure of New Alkaloid Components from *Peganum nigellastrum*. Tennen Yuki Kagobutsu Koen Yoshishu.

[8] Ma Z., Hano Y., Qiu F., Shao G., Chen Y., Nomura T. (2008). Triterpenoids and Alkaloids from the Roots of *Peganum nigellastrum*. Nat. Prod. Commun..

[9] Cagir A., Jones S.H., Eisenhauer B.M., Hecht S.M. (2003). Luotonin A, A Naturally Occurring DNA Topoisomerase I Poison. J. Am. Chem. Soc..

[10] Mussardo P., Corda E., Gonzalez-Ruiz V., Rajesh J., Girotti S., Martin A., Olives A.I. (2011). Study of Non-covalent Interaction of Luotonin A Derivatives and the DNS Minor Groove as a First Step in the Study of Their Analytical Potential as DNA Probes. Anal. Bioanal. Chem..

[11] Wall M.E., Wani M.C., Cook C.E., Palmer K.H., McPhail A.T., Sim G.A. (1966). Plants Antitumor Agents. 1. The isolation and Structure of Camptothecin, a Noval Alkaloidal Leukemia and Tumor Inhibition from *Camptotheca acuminata*. J. Am. Chem. Soc..

[12] Slichemyer W.J., Rowinsky E.K., Donehower R.C., Kaufmann S.H. (1993). The Current Status of Camptothecin Analogs as Antitumor agents. J. Nat. Cancer Invest..

[13] Pommier Y. (1006). Topoisomerase I Inhibitors: Camptothecins and Beyond. Nat. Rev. Cancer.

[14] Pommier Y. (2009). DNA Topoisomerase I Inhibitors: Chemistry, Biology, and Interfacial Inhibition. Chem. Rev..

[15] Pommier Y. (2010). DNA Topoisomerases and Their Poisoning by Anticancer and Antibacterial Drugs. Chem. Biol..

[16] Ma Z., Hano Y., Nomura T. (2005). Luotonin A: A Lead Toward Anti-Cancer Agent Development. Heterocycles.

[17] Huang W.P., Liu J.L., Wang C.L. (2009). Progress in the Synthesis of Natural Product Luotonin A and Its Derivatives. Chin. J. Org. Chem..

[18] Michael J.P. (2008). Quinoline, Quinazoline, and Acridone Alkaloids. Nat. Prod. Rep..

[19] Ma Z.-Z., Hano Y., Nomura T., Chen Y.-J. (1999). Synthesis of Cytotoxic Pyrroloquinazolino-Quinoline Alkaloid Luotonin A. Heterocycles.

[20] Friedländer P. (1882). Über o-Amidobenzaldehyd. Ber. Detsch. Chem. Ges..

[21] Cheng C.-C., Yan S.-J. (1992). The Friedländer Synthesis of Quinolines. Org. React..

[22] Marco-Contelles J., Perez-Mayoral E., Samadi A., Carreiras M. do C., Soriano E. (2009). Recent Advances in the Friedländer Reaction. Chem. Rev..

[23] Kelly T.R., Chamberland S., Silva R.A. (1999). Total Synthesis of Luotonin A. Tetrahedron Lett..

[24] Molina P., Tarraga A., Gonzalez-Tejero A. (2000). A Convenient Divergent Approach to the Alkaloids Isaindigotone and Luoronin A. Synthesis.

[25] Sridharan V., Ribelles P., Terresa Ramos M., Carlos Menendez J. (2009). Cerium(IV) Ammonium Nitrate Is an Excellent, General Catalyst for the Friedländer and Friedländer-Borsche Quinoline Syntheses: Very Efficient Access to the Antitumor Alkaloid Luotonin A. J. Org. Chem..

[26] Mason J.J., Bergman J. (2007). Total Synthesis of Luotonin A and 14-Substituted Analogues. Org. Biomol. Chem..

[27] Harayama T., Morikami Y., Shigeta Y., Abe H., Takeuchi Y. (2003). A Convenient Synthesis of Luotonin A and B. Synlett.

[28] Harayama T., Hori A., Serba G., Morikami Y., Matsumoto T., Abe H., Takeuchi Y. (2004). Concise Synthesis of Quinazoline Alkaloids, Luotonins A and B, and Rutaecarpine. Tetrahedron.

[29] Mhaske S.B., Argade N.P. (2004). Regioselective Quinazolinone-Directed Ortho Lithiation of Quinazolinoylation: Practical Synthesis of Naturally Occurring Human DNA Topoisomerase I Poison Luotonin A and Luotonins B and E. J. Org. Chem..

[30] Chavan S.P., Sivappa R. (2004). A Short and Efficient General Synthesis of Luotonin A, B, and E. Tetrahedron Lett..

[31] Kametani T., Higa T., Loc C.V., Ihara M., Koizmi M., Fukumoto K. (1976). Iminoketene Cycloaddition. 1. A Facile Synthesis of Quinazoline System by Condensation of Iminoketene with Amines – A Total Synthesis of Evodine and Rutaecarpine by Retro Mass-Spectral Synthesis. J. Am. Chem. Soc..

[32] Wang H., Ganesan A. (1998). Total Synthesis of the Cytotoxic Alkaloid Luotonin A. Tetrahedron Lett..

[33] Dallavalle S., Merlini L. (2002). A New Synthesis of the Cytotoxic Alkaloid Luotonine A. Tetrahedron Lett..

[34] Yadav J.S., Reddy B.V.S. (2002). Microwave-assisted Rapid Synthesis of the Cytotoxic Alkaloid Luotonin A. Tetrahedron Lett..

[35] Cagir A., Eisenhauer B.M., Gao R., Thomas S.J., Hecht S.M. (2004). Synthesis and Topoisomerase I Inhibitory Properties of Luotonin A Analogues. Bioorg. Med. Chem..

[36] Cagir A., Jones S.H., Eisenhauer B.M., Gao R., Hecht S.M. (2004). Synthesis and Biochemical Properties of E-Ring Modified Luotonin A Derivatives. Bioorg. Med. Chem. Lett..

[37] Lee E.S., Park J.G., Jahng Y. (2003). A Simple Synthesis of Simple Alkaloids – Syntheis of 2,3-Polymethylene-4(3*H*)-quinazolinones and Related Alkaloids. Tetrahedon Lett..

[38] Jahng K.C., Kim S.I., Kim D.H., Seo C.S., Son J.-K., Lee S.H., Lee E.S., Jahng Y. (2008). One-Pot Synthesis of Simple Alkaloids: 2,3-Polymethylene-4(3*H*)-quinazolinones, Luotonin A, Tryptanthrin, and Rutaecarpine. Chem. Pharm. Bull..

[39] Twin H., Batey R.A. (2004). Intramolecular Hetero Diels-Alder (Povarov) Approach to the Synthesis of the Alkaloids Luotonin A and Camptothecin. Org. Lett..

[40] Zhou H.-B., Liu G.-S., Yao Z.-J. (2007). Short and efficient Total Synthesis of Luotonin A and 22-Hydroxyacuminatine Using A Common Cascade Strategy. J. Org. Chem..

[41] Tangirala R., Antony S., Agama K., Pommier Y., Curran D.P. (2005). Total Synthesis of Luotonin A and a Small Library of AB-Ring Substituted Analogues by Cascade Radical Annulation of Isonitriles. Synlett.

[42] Bowman W.R., Cloonan M.O., Fletcher A.J., Stein T. (2005). Synthesis of Heteroarenes Using Cascade Radical reaction via Iminyl Radicals. Org. Biomol. Chem..

[43] Bowman W.R., Elsegood M.R.J., Stein T., Weaver G.W. (2007). Radical Reactions with 3H-Quinazolin-4-ones: Synthesis of Deoxyvasicinone, Mackinazoline, Luotonin A, Rutaecarpine and Tryptanthrin. Org. Biomol. Chem..

[44] Toyota M., Komori C., Ihara M. (2002). Three-step Total Synthesis of Pyrroloquinazolinoquinoline Alkaloid, Luotonin A, by Intramolecular Hetero Diels-Alder Reaction. Heterocycles.

[45] Toyota M., Komori C., Ihara M. (2003). An Efficient Total Synthesis of Pyrroloquinazolinoquinoline Alkaloid, Luotonin A, Employing An Intramolecular Hetero Diels-Alder Reaction. ARKIVOC.

[46] Servais A., Azzouz M., Lopes D., Courillon C., Malacria M. (2007). Radical Cyclization of N-Acylcyanamides: Total Synthesis of Luotonin A. Angew. Chem. Int. Ed..

[47] Beaume A., Courillon C., Derat E., Malacria M. (2008). Unprecedented Aromatic Homolytic Substitutions and Cyclization of Amide-Iminyl Radicals: Experimental and theoretical Study. Chem. Eur. J..

[48] Ju Y., Liu F., Li C. (2009). Palladium-Catalyzed Sequential Cyanation/*N*-Addition/*N*-Arylation in One-Pot: Efficient Synthesis of Luotonin A and Its Derivatives. Org. Lett..

[49] Tseng M.-C., Chu Y.-W., Tsai H.-P., Lin C.-M., Hwang J., Chu Y.-H. (2011). One-Pot Synthesis of Luotonin A and Its Analogues. Org. Lett..

[50] Wagh M.B., Shankar R., Kumar U.K.S., Gill C.H. (2011). A Concise and Convergent Synthesis of Luotonin B and E. Synlett.

[51] Goller A., Koutentis P.A. (2010). Two-Step synthesis of Canthin-6-one Alkaloids: New One-Pot Sequential Pd-Catalyzed Suzuki-Miyaura Coupling and Cu-Catalyzed Amidation Reaction. Org. Lett..

[52] Soriano-Agaton F., Lagoutte D., Poupon E., Roblot F., Fournet A., Gantier J.-C., Hocquemiller R. (2005). Extraction, Hemisynthesis, and Synthesis of Canthin-6-one Analogue. Evaluation of Their Antifungal Activities. J. Nat. Prod..

[53] Suzuki H., Adachi M., Ebihara Y., Gyoutoku H., Furuya H., Murakami Y., Okuno H.  (2005). Synthetic Studies on Indoles and Related Compounds. Part 53. A Total Synthesis of 1-Methoxycanthin-6-one: An Efficient One-Pot Synthesis of the Canthin-6-one Skeleton from β-Carboline-1-carbaldehyde. Synthesis.

[54] Rossler U., Blechert S., Steckhan E. (1999). Single Electron Transfer Induced Total Synthesis of Canthin-6-one. Tetrahedron Lett..

[55] Mhaske S.B., Argade N.P. (2002). Biogenetic Synthesis of Luotonin F. Synthesis.

[56] Rahman A.F.M.M., Kim D.H., Liang J.L., Lee E.-S., Na Y., Jin K.-Y., Kwon Y., Jahng Y. (2008). Synthesis and Biological Properties of Luotonin Derivatives. Bull. Kor. Chem. Soc..

[57] Narco K., Zha C.C., Guzzo P.R., Herr R.J., Peace D., Friedrich T.D. (2007). Synthesis and Topoisomerase Poisoning Activity of A-Ring and E-Ring Substituted Luotonin A Derivatives. Bioorg. Med. Chem..

[58] Dallavalle S., Merlini L., Beretta G.L., Tinelli S., Zunino F. (2004). Synthesis and Cytotoxic Activity of Substituted Luotonin A Derivatives. Bioorg. Med. Chem. Lett..

[59] Dolubev A.S., Bogomolov V.O., Shidlovski A.F., Dezhenkova L.G., Peregudov A.S., Shtil A.A., Chkanikov N.D. (2010). Synthesis of Fluoromethyl-Containing Analogs of Antitumor Alkaloid Luotonin A. Russ. Chem. Bull..

[60] Lee E.S., Park J.G., Kim S.I., Jahng Y. (2006). Synthesis and Properties of Luotonin A Homologues and Their Aza-analogues. Heterocycles.

[61] Rahier N.J., Cheng K., Gao R., Eisenhauer B.M., Hecht S.M. (2005). Synthesis of 14-Azacamptothecin, a Water-Soluble Topoisomerase I Poison. Org. Lett..

[62] Elban M.A., Sun W., Eisenhauer B.M., Gao R., Hecht S.M. (2006). Synthesis and Biological Evaluation of 10,11-Methylenedioxy-14-azacamptothecin. Org. Lett..

[63] Thomas C.J., Rahier N.J., Hecht S.M. (2004). Camptothecin: Current Perspectives. Bioorg. Med. Chem..

